# Exploring the Intersection of Blood Transfusion and Same-Day Computed Tomography Imaging: An Overview of Clinical Risks and Practices

**DOI:** 10.3390/diagnostics14192201

**Published:** 2024-10-02

**Authors:** Lavinia Alice Bălăceanu, Cristiana Grigore, Cristian-Dorin Gurău, Carmen Giuglea, Gelu-Adrian Popa, Mara Mădălina Mihai, Ion Dina, Beatrice Bălăceanu-Gurău

**Affiliations:** 1 Department of Medical Semiology, “Sf. Ioan” Clinical Emergency Hospital, “Carol Davila” University of Medicine and Pharmacy, 020021 Bucharest, Romania; alice.balaceanu@umfcd.ro (L.A.B.); cristiana.draganescu@drd.umfcd.ro (C.G.); ion.dina@umfcd.ro (I.D.); 2 “Sf. Ioan” Clinical Emergency Hospital, 042122 Bucharest, Romania; 3 Department of Orthopedics and Traumatology, Clinical Emergency Hospital, “Carol Davila” University of Medicine and Pharmacy, 020021 Bucharest, Romania; 4 Orthopedics and Traumatology Clinic, Clinical Emergency Hospital, 014451 Bucharest, Romania; 5 Department of Plastic Surgery, “Sf. Ioan” Clinical Emergency Hospital, “Carol Davila” University of Medicine and Pharmacy, 020021 Bucharest, Romania; carmen.giuglea@umfcd.ro; 6 Plastic Surgery Clinic, “Sf. Ioan” Clinical Emergency Hospital, 042122 Bucharest, Romania; 7 Department of Radiology and Medical Imaging, “Sf. Ioan” Clinical Emergency Hospital, 042122 Bucharest, Romania; 8 Department of Oncologic Dermatology, ”Elias” Emergency University Hospital,” Carol Davila” University of Medicine and Pharmacy, 020021 Bucharest, Romania; mara.mihai@umfcd.ro (M.M.M.); beatrice.balaceanu@rez.umfcd.ro (B.B.-G.); 9 Clinic of Dermatology, “Elias” Emergency University Hospital, 011461 Bucharest, Romania; 10 Research Institute of the University of Bucharest, Department of Botany-Microbiology, Faculty of Biology, University of Bucharest, 050663 Bucharest, Romania; 11 Clinical Department of Gastroenterology, “Sf. Ioan” Clinical Emergency Hospital, 042122 Bucharest, Romania

**Keywords:** transfusion, blood cell component, contrast-enhanced CT, contrast agent

## Abstract

The use of transfusions, whether involving whole blood or specific blood components, is essential for managing various clinical conditions. Many cases are acute, often requiring post-transfusion imaging evaluation. While there is no absolute contraindication for chest imaging following blood transfusion, it should be approached cautiously. We conducted a comprehensive search across multiple databases and registries. Research studies were limited to full-text original articles, reviews, and case reports published in English, involved human subjects, and focused on the interplay between blood transfusions and contrast-enhanced imaging. Scientific analyses were excluded if they did not focus on transfusion practices in the context of imaging or failed to address issues such as hemoglobin thresholds, transfusion reactions, or the clinical implications of contrast agents. Our research fills this gap by emphasizing the need for a cautious, multidisciplinary approach to post-transfusion computed tomography (CT) scans, especially in the presence of contrast agents. This study calls for increased awareness of the heightened risk of complications, such as autoimmune hemolysis, when both procedures are performed together. New insights from our research recommend individualized assessments and close patient monitoring when combining these interventions. Nevertheless, patients need to be hemodynamically and clinically stable before undergoing CT. Discussions. Symptoms that develop within the first 24 h post-transfusion are classified as secondary post-transfusion reactions unless proven otherwise. The prevalence of side effects from same-day CT scans and blood transfusions is challenging to quantify, as few studies focus on this combination. Transfusions and contrast-enhanced CT scans share overlapping adverse reactions and carry significant risks. Acute hemolytic red blood cell transfusion reactions are among the most frequent side effects, with a prevalence of 1:12,000–38,000. Conclusion. Our study contributes new insights to the literature by filling the gap concerning the interplay between transfusions and contrast media, paving the way for more informed clinical protocols to enhance patient safety.

## 1. Introduction

Transfusions of various blood components or whole blood are essential for managing severe clinical conditions. Most clinical trials have not consistently identified potential side effects linked to the hemoglobin threshold; however, variability in reporting methods has hindered quantitative analyses of transfusion reactions in some studies [1]. Our paper aims to explore the clinical implications of different transfusion thresholds, investigate associated mortality and complication rates, and provide evidence-based recommendations to enhance patient outcomes and inform clinical decision-making. Additionally, it addresses the interplay between transfusions and contrast media, contributing new insights that could lead to more effective clinical protocols for improving patient safety.

The ongoing debate between restrictive transfusion (using a hemoglobin threshold of 7 g/dL to 8 g/dL) and liberal transfusion (with a higher threshold) has been extensively studied, particularly about associated side effects and 30-day mortality rates [1]. While restrictive transfusion is frequently recommended, no universally accepted hemoglobin threshold exists for all clinical scenarios [1]. In critical care trials, a threshold of 7 g/dL is commonly used, whereas studies in cardiac and orthopedic surgery tend to adopt higher thresholds [1].

Commonly reported adverse effects include acute or delayed hemolytic transfusion reactions, complications from multiple long-term transfusions, transfusion-related acute lung injury (TRALI), and circulatory overload-induced acute lung injury [2]. Symptoms that manifest within the first 24 h post-transfusion are classified as secondary transfusion reactions until an alternative diagnosis is established [3].

A large study comprising 19 systematic reviews and 33 meta-analyses found no statistically significant difference in mortality between restrictive and liberal transfusion strategies [4]. However, bleeding risk factors may be of particular importance in certain clinical conditions [4]. In a separate study involving patients with coronavirus disease 19 (COVID-19) who required transfusion, a direct correlation was observed between red blood cell transfusion and mortality [5]. Given the complexity of comorbidities in patients with severe COVID-19, further data on diagnoses, laboratory results, and medication use is required to better understand the relationship between transfusion and mortality in this population [5].

Transfusion monitoring encompasses both acute reactions (occurring within the first 24 h) and delayed reactions (emerging more than 24 h post-transfusion) [2]. The initial presentation of severe adverse reactions may resemble that of mild side effects, necessitating a standardized management approach [3]. Some situations are often acute and may escalate to urgent, frequently necessitating further imaging investigations, including post-transfusion evaluations [3].

## 2. Materials and Methods

A comprehensive literature search was conducted to identify relevant studies examining blood transfusion practices, particularly about the use of contrast-enhanced imaging. The search was carried out across multiple databases and registries, including Web of Science Core Collection, PubMed, Google Scholar, Elsevier ScienceDirect, and the Cochrane Database, as well as the websites of International Societies of Transfusion and Radiology. The search aimed to capture a wide range of full-text articles, reviews, and case reports.

The initial search was conducted in December 2023, and updates were performed monthly. Only articles published within the last 10 years (from 1 January 2014 to 25 June 2024) were considered to ensure that the data reflected the most current clinical practices and findings. 

The search employed a combination of medical terms and free-text keywords to maximize the retrieval of relevant studies. Key terms used in the search included “blood transfusion,” “cell component blood transfusion,” “computed tomography (CT),” “CT scan,” “contrast-enhanced CT,” “contrast agent,” and “contrast media.” The selection of search terms was carefully considered to ensure comprehensive coverage of relevant literature while maintaining a focus on the intersection of blood transfusion practices and contrast-enhanced imaging. Given the potential for interaction between transfusions and contrast media, these terms were included to identify studies that specifically address the risks or complications associated with using contrast agents in patients receiving blood transfusions. All terms were chosen to ensure that the search would retrieve studies that address both the clinical practice of transfusions and the potential complications related to contrast-enhanced imaging, such as the timing of imaging procedures or the risk of transfusion-related reactions in the context of contrast media use.

**Figure 1:**
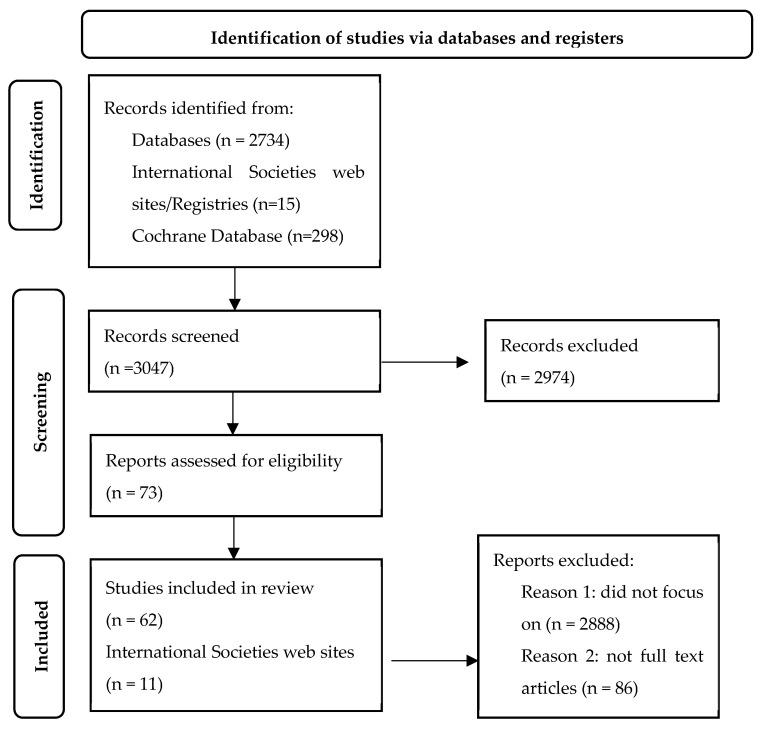
The flowchart underlines the studies included in our research [6].

To ensure the relevance and quality of the studies, several inclusion and exclusion criteria were employed. Studies were limited to full-text original articles, reviews, and case reports published in English, involved human subjects, and focused on the interplay between blood transfusions and contrast-enhanced imaging (Figure 1). Only studies published within the last ten years were included. Studies were excluded if they did not focus on transfusion practices in the context of imaging or failed to address issues such as hemoglobin thresholds, transfusion reactions, or the clinical implications of contrast agents (Figure 1). Non-full-text articles, abstracts, conference proceedings, and animal studies were also excluded.

Data extraction was performed systematically using a predefined data extraction rationale developed specifically for this study. Our rationale was to capture all relevant information from the selected articles, ensuring consistency and comprehensiveness. Studies were evaluated for the clarity of their research questions, the comprehensiveness of the literature search, and the use of appropriate methods for synthesizing data. The extracted data were further organized in Microsoft Excel (version 16.78.3) for tabulation and synthesis.

## 3. Results

Various guidelines provide comprehensive criteria for blood product administration, along with potential adverse reactions and their management. However, they lack specific recommendations regarding the avoidance of contrast-enhanced computed tomography (CT) imaging immediately post-transfusion (Table 1). 

Following transfusion of various blood cell components, both acute and chronic reactions may arise, potentially complicating imaging assessments conducted via contrast-enhanced CT (Table 2).

### 3.1. Acute Transfusion Reactions

Acute transfusion reactions, excluding those caused by ABO red cell incompatibility, can be classified as allergic (e.g., anaphylaxis, urticaria), immune-mediated (e.g., nonhemolytic febrile reactions, transfusion-related acute lung injury), or volume-related (e.g., acute pulmonary edema due to hypervolemia) [2].

- Allergic Reactions

Allergic reactions, such as urticaria and angioedema, occur in 1% to 3% of transfusions, whereas anaphylaxis is rarer, with an incidence of approximately 1 in 20,000 to 50,000 transfusions [2]. A large-scale study conducted over five years, encompassing 8.34 million transfusions, reported an overall prevalence of transfusion-related adverse reactions of 220 per 100,000 components transfused [22]. Among these, allergic reactions were the most common, accounting for 41% of the total, with 9% of these classified as severe or life-threatening [22]. Allergic reactions occur when the recipient’s immunoglobulin E (IgE) antibodies react with plasma protein antigens present in the transfusion product [14]. All blood components, except washed blood, have the potential to trigger allergic reactions, as even small amounts of plasma proteins are sufficient to provoke an immune response [14].

- Acute Intravascular Hemolysis

Acute intravascular hemolysis primarily occurs due to ABO incompatibility or physical and chemical damage to erythrocytes [2,14]. Acute hemolytic transfusion reactions are observed in approximately 1 out of 12,000 to 38,000 red blood cell transfusions, with fatal outcomes reported in 1 per 600,000 to 1.5 million cases [2]. Post-transfusion hemolysis can also arise from non-immune mechanisms, such as osmotic disruption (infusion of hypo-osmolar solutions like dextrose), thermal injury (transfusion of overheated or frozen blood products), or mechanical causes (rapid transfusions or the use of narrow leukodepletion filters) [2,14]. Certain hemolytic reactions can be associated with hemolytic drugs or added components [2,14].

- Transfusion-Related Acute Lung Injury (TRALI)

TRALI is reported at a rate of approximately 1 per 480,000 red blood cell transfusions [2]. In a large-scale study spanning five years and 8.34 million transfusions, pulmonary complications accounted for 35% of severe reactions and 65% of transfusion-related fatalities [22]. TRALI is characterized by respiratory failure resulting from non-cardiogenic pulmonary edema and can occur following transfusion of both red blood cells and platelets [13,19].

There are two distinct types of TRALI, with the CT imaging appearance varying depending on the early or late phase of the condition:

○ Type I:
  Type I TRALI manifests acutely within the first 6 h post-transfusion and is characterized by bilateral pulmonary edema on imaging in the absence of risk factors for acute respiratory distress syndrome (ARDS) or hypertensive pulmonary edema [2,13]. In its early phase, CT typically reveals posterior consolidations (depending on patient positioning) and disseminated ground-glass opacifications, which are symmetrical and similar to ARDS of extrapulmonary origin [23].
○ Type II:
  Type II TRALI presents with similar imaging findings but occurs in patients with pre-existing risk factors for ARDS, although they maintain a stable respiratory status for at least 12 h prior to transfusion [13]. In the late phase, CT findings include ground-glass opacifications in the anterior lung regions, independent of patient positioning, as well as lung cysts and, in cases of invasive ventilation, the presence of air bubbles [23].

- Transfusion Associated Circulatory Overload (TACO)

Acute pulmonary edema may also result from circulatory overload, a condition known as TACO, which occurs in approximately 1% to 8% of transfusions [2,19]. According to NICE guidelines, TACO is the leading cause of transfusion-related mortality [9]. Pulmonary complications from both TACO and transfusion-related acute lung injury are significant contributors to transfusion-related fatalities, as noted by other studies [24]. The excessive volume of transfused blood components or rapid infusion rates leads to acute pulmonary edema in TACO cases [2]. On chest radiography, TACO typically presents as bilateral pulmonary interstitial infiltrates [25]. A thorough assessment of pretransfusion comorbidities—particularly conditions that elevate the risk of TACO, such as hypervolemia, heart failure, hypoalbuminemia, and renal failure—along with adjusting the transfusion rate can significantly reduce the risk of TACO [2,26].

**Figure 2:**
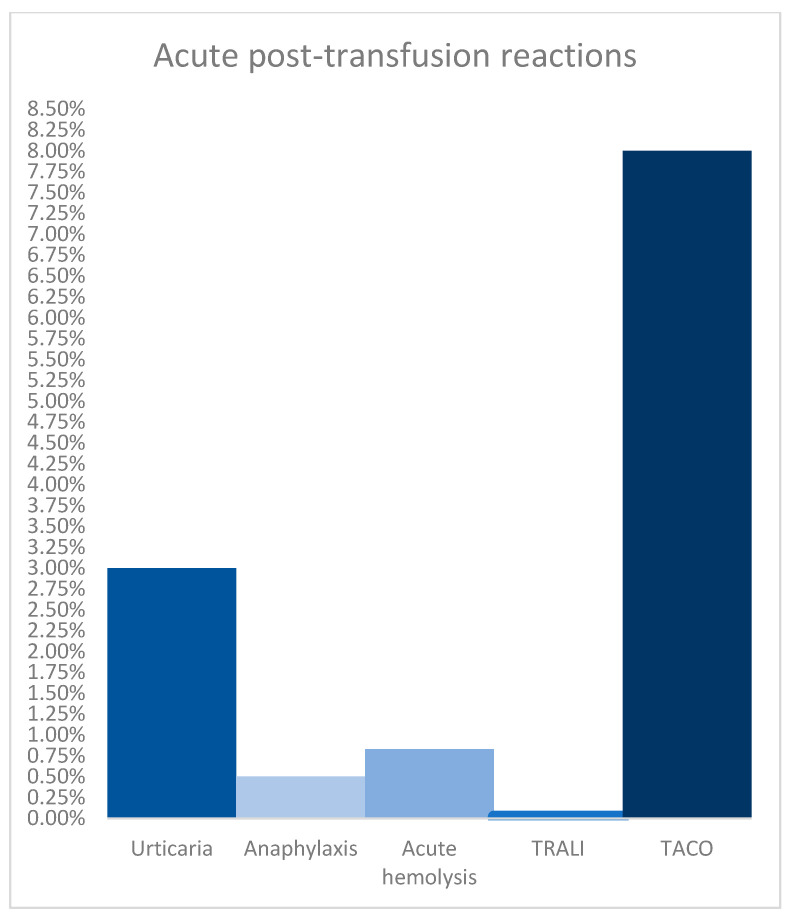
Prevalence of the most common acute post-transfusion reactions: urticaria and angioedema 1–3%, anaphylaxis 1 per 20,000 cases, acute hemolytic transfusion reaction 1 per 12,000 cases, TRALI 1 per 480,000 cases, and TACO 8% [2].

To conclude, allergic reactions and TACO are more common than other acute post-transfusion reactions (Figure 2). These complications may interfere with the administration of contrast agents during CT imaging evaluations.

### 3.2. Delayed Transfusion Reactions 

Apart from iron overload, delayed transfusion reactions are predominantly immune-mediated, including delayed hemolytic reactions, human leukocyte antigen (HLA) alloimmunization, transfusion-associated graft-versus-host disease, and immune responses related to pregnancy [2,15,27].

- Delayed Hemolytic Transfusion Reactions

Delayed hemolytic transfusion reactions are caused by an anamnestic immune response, where previously sensitized antibodies react to transfused red blood cells [27]. Hemolysis primarily occurs extravascularly, and the clinical symptoms, such as anemia and jaundice, are typically mild and rarely severe [27]. These reactions are usually present between 5 and 10 days but can occur up to a month after transfusion [7,27]. The incidence is reported at approximately 1 per 5400 to 62,000 transfusions, with fatal outcomes occurring in 1 per 1.8 million transfusions [2].

- HLA Alloimmunization

Alloimmunization to HLA can sometimes lead to post-transfusion reactions, particularly in patients undergoing multiple transfusion therapies [28]. The prevalence of HLA alloimmunization is reported in 10% to 17% of transfusion recipients with a history of frequent transfusions [2].

- Transfusion-Associated Graft-Versus-Host Disease (TA-GvHD)

TA-GvHD is a rare but severe complication that occurs when non-irradiated blood components are transfused, particularly in immunocompromised patients [2]. This condition usually develops 3 to 30 days post-transfusion and manifests as bone marrow aplasia [2]. TA-GvHD is most commonly associated with transfusions containing viable lymphocytes, which attack the recipient’s tissues [2]. Immunocompromised individuals, such as those undergoing allogeneic or autologous hematopoietic stem cell transplantation, Hodgkin lymphoma patients, or those receiving purine analog treatments or CAR-T cell therapies, are at greatest risk [16]. In these cases, transfusion with irradiated blood components is essential to prevent this often fatal complication [16].

Both gamma and X-ray irradiation of red blood cells raise extracellular potassium levels, so hyperkalemia should be monitored and managed during rapid transfusions, particularly in cases of massive hemorrhage [16]. Blood components that require irradiation include red blood cells, platelets, and granulocytes, while cryopreserved red cells (after deglycerolization), cryoprecipitate, fresh frozen plasma, and fractionated plasma do not need irradiation [16].

### 3.3. Multiple Long-Term Transfusions 

Severe hemolytic reactions can also occur in patients receiving long-term, repeated transfusions, such as those with thalassemia, sickle cell disease, or malaria [27]. The exact pathophysiological mechanisms behind these reactions remain unclear, but they can manifest either acutely or delayed [27]. It is hypothesized that transfused exogenous antigens may induce a pseudo-autoimmune state through leukocyte-driven inflammasome activation [27]. In patients with sickle cell disease, delayed hemolytic transfusion reactions, with or without hyperhemolysis, are frequently observed [29]. These reactions can involve both antigen-negative and antigen-positive red blood cells, often accompanied by complement activation [29]. The use of standard blood filters helps to reduce the incidence of post-transfusion reactions by removing blood clots, coagulated proteins, and cellular debris [20]. In Canada, all blood components undergo filtration during preparation to reduce leukocyte content and must be administered within 4 h [20]. However, no consensus exists on the optimal blood storage duration before transfusion [30,31].

### 3.4. Transfusions in Hematological Diseases

In certain hematological conditions, such as DiGeorge syndrome, congenital immunodeficiencies, allogeneic bone marrow transplantation, and Hodgkin’s lymphoma, patients requiring transfusions are given irradiated blood products to prevent TA-GvHD [18]. Red blood cell components may be irradiated between 14 and 28 days after collection to reduce the risk of this complication [16,28]. Irradiated cellular blood components should ideally be transfused within 14 days after irradiation and no later than 28 days post-harvesting [16,32].

### 3.5. Adverse Reactions Related to the Storage Period

Several studies have reported an increased incidence of adverse reactions in cardiac surgery patients who received red blood cell transfusions with storage periods exceeding 14 days compared to those transfused with cells stored for less than 14 days [13]. Notably, this study excluded patients undergoing massive transfusions, exchange transfusions, or those with severe hemoglobinopathies [13]. A subsequent study found no significant differences in outcomes between patients who received red blood cell transfusions stored for less than 14 days versus those stored for more than 20 days before cardiac surgery [33]. However, the authors were unable to make definitive conclusions regarding blood stored near the maximum recommended limit of 42 days [33]. The 2016 guidelines from the Association for the Advancement of Blood and Biotherapies (AABB) recommend using blood products stored within the licensed dating period [10].

Understanding both acute and delayed transfusion reactions is essential for improving patient safety and outcomes in transfusion medicine. While acute reactions often manifest immediately, they can lead to severe complications if not promptly recognized and managed. Delayed reactions, though typically less acute, can still have significant implications for patient health, particularly in immunocompromised individuals or those requiring long-term transfusions. Continuous education and awareness of the signs, symptoms, and management protocols for both types of reactions are essential for healthcare providers.

Furthermore, implementing best practices in transfusion protocols, including monitoring storage times and considering irradiation for at-risk populations, can mitigate the risks associated with blood transfusions. The role of multidisciplinary teams in evaluating the necessity of transfusions, especially in the context of imaging procedures involving contrast agents, is paramount to ensuring patient safety and minimizing risks. Overall, tailored transfusion practices that prioritize both immediate clinical needs and long-term patient welfare are essential in optimizing therapeutic outcomes. Collectively, these measures will enhance transfusion safety and improve overall patient care.

## 4. Discussion

In current clinical practice, there are situations where blood cell transfusions and CT imaging may both be required on the same day.

### 4.1. Trauma

#### 4.1.1. Transfusion Management in Trauma

Trauma and hemorrhagic shock are significant causes of mortality. In cases of hemorrhagic shock, active bleeding, or hemoglobin levels dropping below 7 g/dL, standard management includes resuscitation, the administration of isotonic crystalloid solutions, and allogeneic red blood cell transfusions [12]. In critically ill patients with traumatic brain injury and anemia, maintaining a hemoglobin threshold above 7 g/dL through liberal transfusion strategies has not been shown to improve neurological outcomes at six months [34].

In cases of severe trauma accompanied by cardiac arrest, circulation-first resuscitation—prior to invasive airway management—may be the initial priority [35]. Pre-hospital transfusions can prevent hypovolemic shock and mitigate the triad of acidosis, coagulopathy, and hypothermia [36,37]. According to one study, the rate of pre-hospital transfusions ranges between 0.2% and 4.4% of patients [36]. However, European guidelines for post-trauma care do not provide specific recommendations for or against pre-hospital blood product administration due to conflicting data and financial considerations [38].

Patients experiencing hemorrhagic shock or with an identifiable source of post-traumatic bleeding require immediate bleeding control [39]. Massive resuscitation carries the risk of serious complications, including ARDS, coagulopathy, abdominal compartment syndrome, and multi-organ failure [40]. To mitigate pulmonary injury and limit coagulopathy, these patients benefit from rapid hemostasis achieved through balanced resuscitation methods and surgical control of bleeding [40]. A ten-year study of patients with severe trauma found exsanguination to be the leading cause of early mortality, with a reported 48% mortality rate in cases involving massive transfusions [41]. Late mortality was predominantly linked to brain trauma (52%) and multiple organ dysfunction (45%) [40]. Specific protocols exist for blunt force and penetrating injuries, addressing blood transfusions and monitoring for acute post-transfusion reactions at 6 h and 24 h intervals [42].

For patients with severe post-traumatic hemorrhage admitted to the emergency department, there is no universally optimal transfusion strategy [43]. Most often, a personalized approach involving a multidisciplinary team is required [43]. These patients typically need a balanced transfusion ratio of plasma, red blood cells, and platelets [43,44]. Emergency physicians may administer blood products in a partial ratio of plasma to red blood cells (1:2), depending on subjective or objective factors such as blood bank availability [43].

European guidelines for post-trauma management emphasize the importance of regularly monitoring bleeding markers, such as hemoglobin and hematocrit, since early phases of hemorrhage may present with normal blood counts [38]. Hemorrhagic shock is a rapidly evolving emergency, and hemoglobin levels alone are not sufficient to determine the need for transfusion to maintain adequate tissue oxygenation [38].

In trauma patients requiring orthopedic surgery, transfusion thresholds are generally set at 8 g/dL in most studies [45,46]. A meta-analysis of trials involving trauma patients undergoing definitive fixation or joint replacement for hip, pelvic, and long bone fractures revealed no acute post-transfusion reactions within the first 24 h following transfusion [46]. Conversely, in trials involving elective hip or knee surgeries, acute post-transfusion reactions within the first 24 h were reported [46].

The most severe transfusion-related complication in emergencies is the administration of blood from an incompatible ABO group, which poses a significant risk [8,47]. The likelihood of clinically significant delayed hemolytic reactions post-transfusion is relatively low, below 0.02% in trauma patients, and is primarily associated with the presence of red blood cell alloantibodies, especially in multiparous women [48].

#### 4.1.2. Imaging Considerations in Trauma: Guidelines and Protocols

In cases of thoracoabdominal trauma, European guidelines advocate for pre-hospital ultrasonographic (PHUS) evaluation to assess for hemothorax, pneumothorax, hemopericardium, and free abdominal fluid [38]. Additionally, whole-body CT is recommended to identify potential sources of bleeding [38]. The benefits of whole-body CT in major trauma patients include facilitating diagnosis, identifying bleeding sources, prioritizing interventions when multiple injuries are present, and improving survival outcomes [38]. For trauma patients without an immediately identifiable bleeding source who do not require urgent bleeding control, imaging investigations such as Focused Assessment with Sonography for Trauma (FAST) ultrasound and contrast-enhanced whole-body CT should be conducted promptly [38,39].

The polytrauma protocol typically includes non-contrast CT of the head and neck alongside contrast-enhanced CT of the chest, abdomen, and pelvis for hemodynamically stable patients [39]. However, the role of immediate whole-body CT in hemodynamically unstable patients is debated, with studies showing varying outcomes [45]. Regardless of timing, CT can be performed either in the resuscitation room or an adjacent space to ensure continuous access to the patient for life-saving interventions [49].

In one study, the placement of a CT scanner within the trauma room or immediately adjacent to it, combined with the accuracy of multi-detector CT technology, was associated with shorter bleeding control times, reduced mortality from exsanguination, and the adoption of immediate whole-body CT in initial trauma evaluation protocols [50]. Another study on patients with severe blunt trauma found that early CT scanning in the trauma resuscitation room significantly reduced exsanguination-related mortality within the first 24 h, while early hemostatic interventions (such as surgery or angioembolization) decreased both 24 h and 28-day mortality [51]. However, immediate total-body CT is contraindicated in cases of hemodynamic instability requiring cardiopulmonary resuscitation or urgent surgical intervention to prevent imminent death [49].

### 4.2. Transfusion Management in Non-Traumatic Critical Patients

In critically ill non-traumatic patients, including those with or without ARDS, current guidelines advocate for restrictive transfusion practices with thresholds set below 7 g/dL [8]. In contrast, elective surgical procedures typically employ a higher transfusion threshold, with hemoglobin levels ranging from 6 to 10 g/dL [52]. Most protocols recommend administering a single unit of blood followed by re-evaluation before proceeding with additional units [52]. Given the potential short- and long-term risks associated with transfusions, decisions regarding intraoperative transfusions should be dynamic and involve a multidisciplinary approach [52].

In elective surgeries, the use of cell salvage techniques can help mitigate the need for perioperative allogeneic blood transfusions [53]. However, some studies suggest that cell salvage may be linked to coagulopathy and increased postoperative bleeding, particularly in cardiac surgeries [53]. Additionally, plasma transfusions, whether given preventively or therapeutically to manage bleeding, carry risks of secondary allergic reactions, lung injuries (such as transfusion-related acute lung injury or transfusion-associated circulatory overload), and thromboembolic complications, including deep vein thrombosis, pulmonary embolism, myocardial infarction, stroke, and disseminated intravascular coagulation [54]. The routine use of prophylactic plasma transfusions prior to non-cardiac surgical interventions in the absence of active bleeding remains a contested topic [11]. Moreover, efforts to identify alternative methods for controlling bleeding and reducing the reliance on transfusions in patients undergoing major vascular procedures have yielded insufficient evidence from clinical trials to substantiate the efficacy of systemic or topical treatments in reducing 30-day mortality, the necessity for surgical reinterventions due to bleeding, or the volume of transfused units [55].

### 4.3. Transfusion Management in Malignancies

In oncological patients with solid or hematological tumors accompanied by anemia, as well as in critically ill individuals suffering from acute neurological injuries, those on ECMO, and elderly patients, the decision to administer either restrictive transfusions below 7 g/dL or liberal transfusions with a threshold of 9 g/dL will be made on an individual basis [1,8,52,55,56]. It is noteworthy that red blood cell transfusions have been linked to increased mortality from various causes and extended hospitalization periods in both oncological patients and those infected with COVID-19 [5,56].

### 4.4. Transfusion Management in Acute Coronary Syndrome

In critically ill patients with acute coronary syndrome, liberal transfusions are advised at a threshold of 9–10 g/dL [8].

### 4.5. Transfusion Management in Sickle Cell Disease

The clinical context becomes particularly complex when acute chest syndrome, characterized by a new infiltrate observed on chest radiography alongside respiratory symptoms, arises in patients with sickle cell disease [57]. In clinical practice, the administration of chronic transfusions may help decrease the incidence of this syndrome [57]. Furthermore, preoperative red blood cell transfusions can serve as a preventative measure against acute chest syndrome in patients with sickle cell disease who are classified as having moderate surgical risk [58]. The decision to administer blood products is contingent upon the specific circumstances of each patient and the clinician’s expertise [57]. While chronic red blood cell transfusions lower the likelihood of primary or secondary stroke in pediatric patients with sickle cell disease, they concurrently elevate the risk of alloimmunization [59].

### 4.6. Transfusion Management in Gastro-Intestinal Bleeding

Restrictive transfusion strategies, maintaining hemoglobin levels below the 7 g/dL threshold, have been shown to enhance survival rates in patients experiencing acute upper gastrointestinal bleeding linked to peptic ulcers, as well as those with Child-Pugh class A and B cirrhosis [60,61]. Notably, for patients with acute bleeding resulting from variceal rupture, a restrictive transfusion approach correlates with improved six-week survival rates and a reduced incidence of re-bleeding compared to more liberal transfusion practices [17]. Liberal transfusion strategies have also been associated with significantly elevated portal pressures [17]. In scenarios involving massive hemorrhage, the administration of uncross-matched blood to patients with undiagnosed red blood cell alloantibodies poses a risk for delayed hemolytic transfusion reactions [21].

### 4.7. Imaging Considerations in Nontraumatic Emergency

In emergencies, imaging studies such as CT, particularly with the use of contrast agents, may be essential for enhanced visualization of blood vessels and tissues. In cases of diverticular bleeding, early administration of contrast-enhanced CT can effectively identify the source of hemorrhage in patients who are unable to undergo bowel preparation and colonoscopy or whose hemodynamic status does not permit endoscopic evaluation [62]. The contrast agents typically utilized are iodine-based. Although autoimmune intravascular hemolysis induced by contrast agents is a rarely documented complication in the literature, it is associated with a poor prognosis [63,64,65,66]. This condition is characterized by the presence of immunoglobulins, as indicated by a positive direct antiglobulin test [67]. Consequently, when a patient who has received a transfusion requires a contrast-enhanced CT examination, it is imperative to ensure that there are no contraindications or adverse reactions to the specific contrast agent employed [67]. Contemporary iodinated or gadolinium-based contrast agents infrequently provoke allergic reactions, typically in patients with pre-existing conditions [67]. Among hematological disorders necessitating frequent transfusions, sickle cell disease or sickle cell trait may trigger an acute sickle crisis in response to the contrast agent [67]. However, there is insufficient evidence to suggest that this reaction also occurs with modern iodinated or gadolinium-based contrast agents [67]. Therefore, the use of these contrast agents is not inherently contraindicated in individuals with sickle cell disease or trait [68].

### 4.8. Guidelines in Contrast-Enhanced CT Examination

Numerous guidelines delineate the criteria for conducting imaging examinations utilizing contrast agents alongside potential adverse reactions and their management strategies [68,69,70,71,72]. However, these guidelines do not specifically address the indications or contraindications for the administration of contrast media in the immediate post-transfusion period (Table 3). Transfusions may lead to hemodilution, resulting in a decreased concentration of red blood cells in the bloodstream, which can adversely affect the quality of images obtained via CT. Nevertheless, modern imaging equipment is equipped to adjust technical parameters in response to variations in blood composition. Furthermore, the patient needs to be hemodynamically and clinically stable following transfusion before being transported to the CT department [69].

In summary, the prevailing threshold for transfusion in most clinical scenarios is a hemoglobin level of 7 g/dL, thereby supporting a restrictive transfusion strategy. For traumatic patients undergoing orthopedic surgery and those with acute coronary syndrome, the threshold is set at 8 g/dL hemoglobin. The majority of secondary reactions associated with blood component transfusions are acute, primarily involving allergic, hemolytic, or pulmonary edema responses. In certain instances, ultrasonographic evaluations may yield inconclusive results, necessitating a contrast-enhanced CT assessment on the same day as the transfusion. Critical or urgent clinical situations—such as major trauma, gastrointestinal hemorrhages, and cardiovascular or abdominal emergencies—may further complicate the decision-making process. Given the allergic potential of contrast agents and the risk of immune hemolysis, the determination to proceed with tomographic evaluation using contrast agents should be made by a multidisciplinary medical team, aiming to maximize diagnostic information while minimizing associated risks.

### 4.9. Future Perspectives

Further studies are needed to assess the long-term outcomes of patients receiving multiple transfusions, particularly in populations with chronic conditions such as sickle cell disease, thalassemia, or malignancies. Understanding the implications of repeated exposure to transfused blood products on immune response and overall health will be essential.

While current guidelines suggest specific hemoglobin thresholds for transfusion, research could explore the optimal thresholds in various patient populations, including those with acute coronary syndrome, trauma, or post-operative recovery. As transfusion practices continue to evolve, research could focus on understanding the variability in adherence to transfusion guidelines among healthcare providers. Identifying barriers to guideline implementation and exploring strategies for improving compliance will enhance patient safety and outcomes. Additionally, the impact of individual patient characteristics on transfusion requirements should be investigated. There is also a need for more rigorous comparisons of the effectiveness and safety of various blood components (e.g., red blood cells, platelets, plasma) across different clinical scenarios. Future research could address the benefits and risks associated with using leukocyte-reduced versus non-leukocyte-reduced products [73,74].

The immunomodulatory effects of transfusions remain an area of active investigation. Future studies could focus on the mechanisms by which transfused blood products affect immune function, including the role of donor characteristics, storage conditions, and pre-transfusion processing techniques [73,74]. Given the pandemic’s impact on healthcare practices, research is therefore needed to understand how COVID-19 has influenced transfusion medicine, including changes in donor behavior, transfusion protocols, and patient outcomes [73,74].

The advent of new technologies, such as point-of-care testing and improved blood storage methods, warrants investigation into their impact on transfusion practices. Research could assess how these innovations affect transfusion decisions, patient outcomes, and overall healthcare costs.

As transfusion practices evolve, there is a need for ongoing dialogue about the ethical implications of transfusions, particularly concerning consent, the allocation of resources, and the management of donor blood supply in the face of shortages [73,74]. Future studies could focus on the investigation of patients’ preferences and perceptions regarding transfusion, including the risks and benefits, which can inform shared decision-making in transfusion practices. Research into patient education interventions could also help demystify transfusions for patients and their families [73,74].

Incorporating these areas into future research can help address critical gaps in knowledge and improve transfusion practices, ultimately enhancing patient care and safety.

## 5. Conclusions

In conclusion, the management of blood transfusions and imaging procedures in critically ill patients requires a nuanced and multidisciplinary approach. The evolving guidelines highlight the importance of individualized transfusion thresholds, particularly in complex clinical scenarios such as acute gastrointestinal bleeding, trauma, and hematological disorders. A restrictive transfusion strategy typically set below 7 g/dL is often associated with improved outcomes, while specific patient populations, including those undergoing orthopedic surgery or experiencing acute coronary syndrome, may necessitate higher thresholds. The interplay between transfusion and subsequent imaging, particularly with contrast agents, underscores the need for careful assessment of patient stability and the potential for adverse reactions. As healthcare professionals navigate these challenging decisions, a collaborative effort involving hematologists, radiologists, and emergency care teams is essential to optimize patient safety and clinical efficacy. Ultimately, fostering an environment of rigorous clinical evaluation and adherence to evidence-based guidelines will enhance patient outcomes and reduce complications, paving the way for advancements in transfusion medicine and critical care practices.

## Figures and Tables

**Table 1:** International societies and guidelines on red blood cell transfusion.

Guidelines	Year	References
European Committee (partial agreement on blood transfusion EDQM	2023	[7]
European Society of Intensive Care medicine (ESICM)	2020	[8]
NICE	2015	[9]
AABB	2016, 2023	[10,11]
EAST/ACCM	2009	[12]
American Red Cross	2021	[13]
American Society of Anesthesiologists	2015	[14]
British Society for Haematology (BSH)	2012, 2020, 2022	[15,16,17]
Association of Anesthetists of Great Britain and Ireland (AAGBI)	2016	[18]
Canadian Blood Services	2019, 2020, 2021	[19,20,21]

**Table 2:** Acute and chronic transfusion-related side effects.

Acute Transfusion Reactions	Delayed Transfusion Reactions
Allergic Reactions	Delayed Hemolytic Transfusion Reactions
Acute Intravascular Hemolysis	HLA Alloimmunization
Transfusion-Related Acute Lung Injury (TRALI)	Transfusion-Associated Graft-Versus-Host Disease (TA-GvHD)
Transfusion Associated Circulatory Overload (TACO)	

**Table 3:** International societies and guidelines on CT with contrast media.

Guidelines	Year	References
American College of Radiology (ACR Manual on Contrast Media)	2023	[68]
European Society of Emergency Radiology	2020	[69]
Japan Radiological Society	2021	[70]
Australian Society of Medical Imaging and Radiation Therapy	2024	[71]
Royal College of Radiologists	2024	[72]
